# Lipid-lowering agents in solid organ transplant recipients

**DOI:** 10.1093/ndt/gfaf104

**Published:** 2025-06-17

**Authors:** Agnieszka Mickiewicz, Sławomir Żegleń, Karolina Kędzierska-Kapuza, Zbigniew Heleniak, Anna Frankiewicz, Ewa Adamczyk, Marcin Wełnicki, Marta Wawrzynowicz-Syczewska, Joanna Raszeja-Wyszomirska, Magdalena Durlik, Maciej Banach, Alicja Dębska-Ślizień, Marcin Gruchała, Jolanta Malyszko

**Affiliations:** First Department of Cardiology, Medical University of Gdansk, Gdansk, Poland; Department of Pneumonology and Allergology, Medical University of Gdansk, Gdansk, Poland; Department of Internal Medicine, Endocrinology and Diabetology, National Medical Institute of the Ministry of Interior Affairs and Administration, Warsaw, Poland; Department of Nephrology, Transplantology and Internal Medicine, Medical University of Gdansk, Gdansk, Poland; First Department of Cardiology, Medical University of Gdansk, Gdansk, Poland; Department of Transplantation Medicine, Nephrology and Internal Diseases, Medical University of Warsaw, Warsaw, Poland; 3rd Department of Internal Diseases and Cardiology at the Medical University of Warsaw Warsaw, Poland; Department of Infectious Diseases, Hepatology and Liver Transplantation, Pomeranian Medical University, Szczecin, Poland; Department of Hepatology, Transplantology, and Internal Medicine, Medical University of Warsaw, Warsaw, Poland; Department of Transplantation Medicine, Nephrology and Internal Diseases, Medical University of Warsaw, Warsaw, Poland; Department of Preventive Cardiology and Lipidology, Medical University of Lodz, Lodz, Poland; Department of Cardiology and Congenital Diseases of Adults, Polish Mother's Memorial Hospital Research Institute, Lodz, Poland; Ciccarone Center for the Prevention of Cardiovascular Disease, Johns Hopkins University School of Medicine, Baltimore, MD, USA; Cardiovascular Research Centre, Zielona Góra, Poland and; Department of Nephrology, Transplantology and Internal Medicine, Medical University of Gdansk, Gdansk, Poland; First Department of Cardiology, Medical University of Gdansk, Gdansk, Poland; Department of Nephrology, Dialysis and Internal Medicine, Medical University of Warsaw, Warsaw, Poland

**Keywords:** dyslipidaemia, ezetimibe, PCSK9 inhibitors, solid organ transplantation, statins

## Abstract

Although pre- and post-transplant dyslipidaemia is one of the most prevalent modifiable risk factors associated with an increased risk of major cardiovascular events, it remains underdiagnosed and undertreated. Moreover, the risk of cardiovascular events, acute allograft rejection and vasculopathy associated with dyslipidaemia is underestimated. Although the most prominent underlying cause of dyslipidaemia in solid organ transplant (SOT) recipients is immunosuppressants, their adjustment should be done with caution to avoid an acute graft rejection. Dietary intervention and lipid-lowering therapy (LLT) are needed to lower low-density lipoprotein cholesterol (LDL-C) and triglycerides and to improve the outcomes. Although statins are first-line drugs, non-adherence, interactions with immunosuppressants and the concern related to polypharmacy impact statin use in SOT patients. The evolving evidence on combination therapy with statin and ezetimibe, novel PCSK9 modulators and bempedoic acid indicate that LDL-C can be safely and efficiently reduced with improved adherence. Since SOT patients are complex, a structured multidisciplinary team approach can deliver comprehensive lipid management, improve patient care and prevent potential complications. A call to action is needed for further trials and registries to determine potential benefits of strategy based on initial combination therapy with ezetimibe and a low/moderate dose of statin, as well as novel LLT. Optimal lipid treatment targets in SOT recipients should be determined, depending on the transplanted organ and cardiovascular risk category. We aimed to review current and future management of lipid disorders, propose an algorithm useful in clinical practice and call attention to broader use of novel LLTs along with further studies to assess their impact on clinical outcomes.

## INTRODUCTION

Heart, kidney, lung, liver and pancreas transplantation revolutionized the treatment of end-stage failure of these organs. Recent advances in surgical techniques, immunosuppressive treatment and infection therapy have improved post-transplantation survival and revealed cardiovascular disease (CVD) as the leading cause of death in heart transplant (HTx) and kidney transplant (KTx) recipients, and the second leading cause in liver transplant (OLTx) recipients [1]. Dyslipidaemia and CVD diagnosed in the pre-transplant period can impact on graft function and patient survival.

Although in solid organ transplant (SOT) recipients dyslipidaemia is one of the most prevalent modifiable cardiovascular (CV) risk factors, it is often underdiagnosed and undertreated. Lipid disorders affect up to 81% of HTx recipients, 78% of KTx recipients, 51% of OLTx patients and >70% of lung transplant recipients and are often underdiagnosed and undertreated, and more importantly the risk associated with dyslipidaemia is underestimated [1]. Elevated low-density lipoprotein cholesterol (LDL-C) and non-high-density lipoprotein cholesterol (HDL-C) and low HDL-C levels in SOT recipients are associated with an increased risk of major adverse cardiovascular events (MACE) ≈3 times higher than in the general population. Dyslipidaemia after SOT is also associated with graft vasculopathy and graft rejection, particularly in KTx and HTx recipients [2].

Dyslipidaemia covers a broad spectrum of lipid disorders, including hypercholesterolaemia, hypertriglyceridaemia, mixed hyperlipidaemia, low HDL-C and high lipoprotein(a) [Lp(a)] levels. In the first year after a SOT the most prevalent lipid disorder is elevated LDL-C and/or a high triglyceride (TG) concentration [3].

Hypercholesterolaemia in SOT recipients can be caused by genetic predisposition, obesity, older age, excessive intake of saturated fats and cholesterol, proteinuria and therapy with beta-blockers, diuretics, immunosuppressants (IS). Post-transplant hypertriglyceridaemia can be induced by genetic predisposition, alcohol consumption, high carbohydrate intake, diabetes, overweight and obesity, proteinuria, renal failure and IS therapy [mostly glucocorticosteroids (GCS), mammalian target of rapamycin (mTOR) and calcineurin-inhibitors (CNIs)]. Depending on the mechanism of action, IS drugs can increase the levels of total cholesterol (TC), LDL-C, TGs and very-low-density lipoprotein particles and affect the size and density of LDL particles [4].

### Management of dyslipidaemia after SOT

The management of dyslipidaemia after SOT includes lifestyle changes such as dietary intervention, weight reduction and increased physical activity. Before initiating lipid-lowering therapy (LLT) in SOT recipients, attention should be paid to modifiable causes of lipid disorders, the risk of potential side effects and drug interactions with IS medication. In case of therapy with cyclosporin A (CsA), mTOR inhibitors or GCS, it is worth considering a dose reduction or switching to another immunosuppressant.

LLT includes statins and ezetimibe, followed by monoclonal antibodies against proprotein convertase subtilisin/kexin type 9 (PCSK9; alirocumab, evolocumab), inclisiran and lipoprotein apheresis, but with limited data [3]. Hypertriglyceridaemia should be treated with statins, fibrates and omega-3 fatty acids [1].

The first-line LLT in post-transplant patients is statins. The evidence showed that statins improved survival rate, reduced the incidence of graft rejection and decreased the risk of primary graft dysfunction. In HTx recipients, statins reduced the risk of cardiac allograft vasculopathy (CAV) and in KTx recipients they lowered the risk of interstitial fibrosis/tubular atrophy (IF/TA). In lung transplant (LTx) recipients, the use of statins may be associated with a decreased risk of chronic lung allograft dysfunction (CLAD) and bronchiolitis obliterans syndrome (BOS) [5]. SOT recipients may also be treated with ezetimibe. Limited data are available on the use of monoclonal antibodies against PCSK9 (alirocumab, evolocumab), inclisiran and lipoprotein apheresis [6–8]. Adherence to statin treatment remains an obstacle in achieving LDL-C goals. In the general population, only 20% of statin users are still on the drug after 1 year. The main causes are statin-related adverse effects, mainly statin-associated muscle symptoms (SAMS) and the elevation of liver enzymes. In SOT recipients, concern related to the adjustment of IS to avoid an acute graft rejection, common polypharmacy and drug–drug interactions with IS medications further impact statin use. Therefore, combination therapy with statin and ezetimibe as well as novel agents, i.e. PCSK9 modulators, can provide safe and efficient LDL-C reduction [9]. Long-term administration of parenteral evolocumab, alirocumab and inclisiran can safely reduce LDL-C by 49–59%. Moreover, these medications have no pharmacokinetic interferences with IS. Several trials on PCSK9 inhibitors in statin intolerance reported no difference between PCSK9 inhibitor and placebo in terms of muscle-related adverse events.

The evidence suggests that adherence can be further improved with inclisiran, administered every 6 months by healthcare professionals, as this drug gives only mild to moderate injection site reactions and was also investigated in patients with chronic kidney disease (CKD) [10].

A new drug, bempedoic acid (BA), has recently been registered for patients with statin intolerance. As the mechanism of action is related only to hepatic cells, not muscles, BA can be administered orally in patients with a history of SAMS. BA in combination with ezetimibe once daily reduces LDL-C by 40% along with CV events [11].

The evolving evidence in the general population on PCSK9 monoclonal antibodies, inclisiran and bempedoic acid indicates that these drugs would be acceptable with non-adherence in SOT recipients. Despite similar efficacy as in the general population and the low rate of interactions with IS, these medications are still rarely used in this vulnerable population. Future prospective studies and outcomes trials could evaluate the safety and efficacy of novel lipid-lowering drugs in SOT recipients [10, 12–14].

There is a lack of specific recommendations on the management of lipid disorders and sufficient data on novel lipid-lowering agents in SOT recipients. Therefore, we aimed to review current and future management of lipid disorders in SOT patients, propose an algorithm for LLT that will be useful in clinical practice and call attention to the broader use of novel LLTs along with further trials and registries to assess their impact on clinical outcomes.

Strategies to manage dyslipidaemia in SOT recipients with adjustment of diet and statins are insufficient. Although IS drugs are the common cause of new-onset dyslipidaemia or exacerbation of previous lipid disorders, they are crucial in reducing the risk of transplant rejection. Among IS medications, GCS, CNIs and mTOR inhibitors affect the lipid profile. Sirolimus and everolimus cause mainly hypertriglyceridaemia [1, 4]. It is worth noting that CsA increases the blood concentration of all statins and the risk of myopathy [1, 4]. The strategy of reducing GCS doses or early withdrawal improves the lipid profile, but it should be done with caution to avoid graft rejection. Considering the risk of graft rejection, complications of IS therapy and potential drug interactions, the individualization of IS therapy is extremely important after SOT [1].

Diet intervention is a part of lifestyle modification in dyslipidaemia in SOT recipients. The most beneficial dietary patterns in the SOT population are the Mediterranean and Dietary Approaches to Stop Hypertension (DASH) diets. The Mediterranean-style eating pattern is based on vegetables, fruits, legumes, whole grains, beans, seeds, nuts and olives. Eating fish at least two times per week along with less meat and fewer sweets is encouraged. Eggs, poultry, cheese and yogurt can be eaten in moderation. The DASH diet is a balanced and flexible healthy eating plan, and no special food is recommended, except restriction of sodium to <2.3 g/day. SOT patients should be advised that healthy dietary patterns need to be long term and sustainable [15].

Diet is a fundamental part of the treatment of hypertriglyceridaemia. TG levels can be reduced with the adjustment of carbohydrate, fat and protein percentages in relation to total energy. It is crucial to decrease the intake of simple carbohydrates from 20–50 g/day initially with further decrease to 120–150 g/per day. The consumption of processed foods high in refined sugar and saturated/trans fats should be avoided, while the eating of functional foods such as fruits, legumes, vegetables and olive oil is encouraged. Caloric restriction and weight loss are also proposed as a strategy to lower lifestyle-related hypertriglyceridaemia. Moreover, a diet enriched with 30 g of mixed nuts, 30 g of whole flaxseed and 25 ml of flaxseed oil may improve TG concentrations in subjects with CV risk. Adding to omega-3 supplements has been shown to reduce TG in a dose-dependent manner [16]. A limited number of studies involving dietary interventions have been reported in OLTx, HTx and KTx recipients. The Mediterranean diet in KTx recipients led to a significant reduction in TC levels by 10%, TG by 6.5% and LDL-C by 10.4%. Dietary intervention in HTx recipients followed for 48 months showed that diet compliance was associated with a decrease in TC and TG and weight loss mainly due to a decrease in fat mass [17]. Higher adherence to a Mediterranean diet was associated with a lower risk of lipid profile abnormalities in KTx recipients. The DASH diet was found to improve TC and LDL-C concentration [15]. Moreover, the DASH and Mediterranean diets were associated with favourable outcomes in slowing renal allograft function decline. Close monitoring during intervention is necessary to individualize nutritional support. Statin use in SOT recipients remains challenging. Statins are first-line drugs in dyslipidaemia after SOT [1], with proven benefits in CV outcome, graft rejection rate and graft vasculopathy incidence in the HTx and KTx cohorts [18, 19]. Statins are now recommended for all HTx [International Society for Heart and Lung Transplantation (ISHLT) Class IA] and KTx [Kidney Disease: Improving Global Outcomes (KDIGO) Class 2A] patients. Only patients <30 years of age without CV risk factors could choose not to receive treatment [20, 21].

The statin with the highest potency in terms of LDL-C reduction is rosuvastatin, providing lower LDL-C levels and comparable efficacy for the composite outcome of all-cause death, myocardial infarction, stroke or any coronary revascularization at 3 years compared with atorvastatin [22].

Nevertheless, rosuvastatin, compared with atorvastatin, was associated with an increased risk of new-onset haematuria and proteinuria. The risk was higher with a higher rosuvastatin dose and lower estimated glomerular filtration rate (eGFR) levels [23].

Although statins are first-line drugs, the common non-adherence, potential interactions with IS and concern related to polypharmacy impact statin use in SOT patients. The use of statins with other CYP3A4 inhibitors (CsA, tacrolimus, macrolides, azoles, fibrates, acetaminophen, calcium channel blockers) may increase the risk of myopathy and toxic liver damage [1, 4]. Possible interactions between statins, IS azoles and macrolides are presented in Table 1. The interaction of statins with CYP3A4-inducing drugs (rifampicin, phenytoin, dexamethasone, phenobarbital, phenytoin) may reduce the effectiveness of statins [4].

**Table 1:** Interactions between lipid-lowering agents, IS medications, fluconazole and macrolides.

Lipid-lowering agent	Cyclosporine	Tacrolimus	Everolimus	Fluconazole	Macrolide
Atorvastatin	Major risk of liver damage and rhabdomyolysis	Moderate risk of rhabdomyolysis	No interaction	Major risk of liver damage and rhabdomyolysis	Moderate risk of rhabdomyolysis
Fluvastatin	Moderate risk of rhabdomyolysis	ND	No interaction	Moderate risk of rhabdomyolysis	No interaction
Lovastatin	Major risk of liver damage and rhabdomyolysis	Moderate risk of rhabdomyolysis	No interaction	Major risk of liver damage and rhabdomyolysis	Major risk of rhabdomyolysis
Pitavastatin	Major risk of liver damage and rhabdomyolysis	ND	No interaction	No interaction	No interaction
Pravastatin	Moderate risk of rhabdomyolysis; maximum 20 mg/day	ND	No interaction	No interaction	No interaction
Rosuvastatin	Moderate risk of rhabdomyolysis; maximum 50 mg/day	ND	No interaction	Minor risk of rhabdomyolysis	Minor risk of rhabdomyolysis
Simvastatin	Major risk of liver damage and rhabdomyolysis	Moderate risk of rhabdomyolysis	No interaction	No interaction	Major risk of liver damage and rhabdomyolysis
Fenofibrate	Moderate risk of kidney injury	ND	Decrease level of everolimus	No interaction	No interaction
Ezetimibe	Moderate risk of kidney injury	ND	No interaction	No interaction	No interaction
Omega-3 fatty acids	No interaction	No interaction	No interaction	No interaction	No interaction
Evolocumab	No interaction	No interaction	No interaction	No interaction	ND
Alirocumab	ND	ND	ND	ND	ND
Inclisiran	ND	ND	ND	No interaction	No interaction
Bempedoic acid	No interaction	No interaction	No interaction	No interaction	No interaction

ND: no data.

The most common SAMS include myalgia (muscle pain), myopathy (muscle damage with an increase in creatine kinase) and rhabdomyolysis (muscle damage with an increase in creatine kinase, often >10 times the upper normal limit, and acute kidney injury), as well as liver damage with an increase in the activity of transaminases. The highest risk of side effects is observed in elderly patients, those receiving high doses of statins and those who use drugs metabolized by CYP3A4, such as CNIs [24]. From a practical point of view, when using high-intensity statins (atorvastatin, rosuvastatin) that lower LDL-C by 50%, tacrolimus may be a safe combination for IS therapy. HTx recipients receiving tacrolimus tolerated high-intensity statins well [3, 26].

Nevertheless, studies in the general population showed significant gaps in the use of recommended first-line treatments. Up to 67% of patients are non-adherent to statins 15 months after treatment initiation. Given the interactions with IS drugs, statin use in SOT recipients remains even more challenging. Thus statins post-transplantation are used with caution and at a lower dosage, translating into an insufficient LDL-C decrease and CVD prevention.

### Strategies beyond statins in SOT recipients

The second-line drug used in hypercholesterolaemia is ezetimibe, which lowers LDL-C by 15–20% [1]. However, if ezetimibe must be used along with CsA, the dosage must be reduced to 5 mg/day, as CsA can induce a 4- to 12-fold increase in ezetimibe concentrations and CsA concentrations can increase by 15%.

The SHARP trial (NCT00125593) reported the efficacy and safety of lowering LDL-C with a combination of ezetimibe and simvastatin among a wide range of patients with advanced CKD without a history of myocardial infarction or coronary revascularization, of whom almost 30% were on dialysis. Compared with either placebo or simvastatin alone, a daily combination of simvastatin 20 mg plus ezetimibe 10 mg yielded average LDL-C differences of 43 mg/dl at 1 year and 33 mg/dl at 2.5 years, without any excess of myopathy, hepatic toxicity or biliary complications [25].

The RACING trial (NCT03044665), which included 3780 patients with atherosclerotic cardiovascular disease (ASCVD), showed that patients on combination therapy with a moderate-intensity statin (rosuvastatin 10 mg) and ezetimibe in a 3-year follow-up more frequently achieved serum LDL-C concentrations <70 mg/dl compared with rosuvastatin 20 mg in monotherapy (72% versus 58%; *P* < .0001). Moreover, combination therapy was linked to substantially lower risk of therapy discontinuation or drug dose reduction (4.8% versus 8.2%; *P* < .0001), while maintaining the same cardiovascular benefits [26].

Studies investigating combined therapy with statin and ezetimibe in SOT recipients have shown its effectiveness and safety in reducing TC, LDL-C and TG [8]. Hypertriglyceridaemia in SOT recipients can be managed with fibrates and omega-3 fatty acids. The KDIGO guidelines in KTx recipients recommend including fibrates at a TG level >500 mg/dl. However, before fibrates initiation, excessive alcohol use and impaired glucose control should be excluded or modified. Checking TG levels in a fasting state at least once more should be carried out.

In cases of elevated LDL-C and TG levels of 200–500 mg/dl, intensification of statins and consideration of a fibrate in the next stage is recommended. In post-transplant patients on mTOR inhibitor therapy who experience an increase in TG >500 mg/dl, dietary intervention should be used first. In addition, a reduction in the dosage of mTOR inhibitors or a change in the IS therapy regimen should be considered.

In the case of persistent TG levels >1000 mg/dl, fibrates should be started immediately due to the increased risk of acute pancreatitis and consideration should be given to possible adjustment of IS therapy [1].

Nevertheless, adding fibrates to a statin is associated with the risk of interactions and muscle pain. In addition, an increased graft rejection rate and graft failure were reported for fenofibrate [27].

Omega-3 fatty acids, including eicosapentaenoic acid (EPA) and docosahexaenoic acid (DHA), can be considered as a safe alternative in post-transplant hypertriglyceridaemia. In studies in post-KTx subjects, omega-3-rich fish oil improved graft function [1].

Although omega-3 fatty acids do not cause significant side effects, an increase in the rate of atrial fibrillation (AF) in the EPA versus placebo group was observed. Recent meta-analyses reported that treatment with EPA + DHA was associated with a 24% increased relative risk of AF, but in a dose-dependent manner, which may be mediated by vagal tone. The doses of DHA + EPA of 1000 mg/day increased AF risk by 12%, whereas doses of 1800–4000 mg/day increased AF risk by ≈50%. In contrast, observational studies focusing on DHA + EPA blood levels or dietary intake reported that higher omega-3 consumption/levels were linked with a decreased AF risk [28].

No significantly increased risk of interaction with IS drugs or increased graft rejection rate was observed for omega-3 fatty acids. Only for CsA have minimal interactions with omega-3 fatty acids been reported.

It is worth noting that EPA and DHA differ in their mechanisms of action. Recent data provided an important conclusion from the REDUCE-IT (NCT01492361) and STRENGTH (NCT02104817) trials. EPA at a dose of 4 g/day combined with a statin in a population with TG levels of 135–499 mg/dl and LDL-C levels of 41–100 mg/dl in REDUCE-IT trial reduced not only TG levels (by 25%), but also the risk of CV events. Nevertheless, DHA + EPA in a dose of 4 g/day in the STRENGTH trial, carried out in individuals with TG levels of 180–500 mg/dl, despite a TG reduction, did not cause a reduction in CV event rates [31, 32]. Currently two products have been approved as prescription drugs: Omacor and Vascepa (containing only EPA).

The value of novel lipid-lowering agents in SOT recipients appears to be increasing [12]. PCSK9 inhibitors are the third line of treatment in hypercholesterolaemia, or an alternative to statins in case of intolerance. These drugs, by blocking the PCSK9 protein, increase the density of LDL receptors on the liver surface and enhance their internalization, resulting in an ≈50–60% reduction in LDL-C [29, 30]. PCSK9 inhibitors also exhibit anti-aggregation, anticoagulation and anti-inflammatory effects, stabilizing atherosclerotic plaque.

The ODYSSEY Outcomes trial (NCT01663402) of patients after acute coronary syndrome (ACS) showed a significant reduction of LDL-C by 54.7% at 48 weeks, translating into a decrease in the risk of recurrent ischaemic cardiovascular events. The incidence of adverse events was similar in the two groups, except for local injection-site reactions (3.8% in the alirocumab group versus 2.1% in the placebo group) [31].

The FOURIER trial (NCT01764633) showed that evolocumab in patients with ASCVD reduced LDL-C by 59% at 48 weeks as well as a composite primary endpoint (cardiovascular death, myocardial infarction, stroke, hospitalization for unstable angina or coronary revascularization) [32]. Moreover, PCSK9 inhibitors can decrease atherosclerotic plaque. As the mechanism of action of PCSK9 inhibitors does not involve CYP3A4, these drugs do not cause drug interactions in patients after SOT [29]. Despite the observed reduction in CsA and sirolimus levels, there was no increased risk of graft rejection. Meta-analysis of nine studies involving 110 patients after HTx showed that alirocumab and evolocumab were well tolerated and lowered LDL-C by 40–87% [29]. Recently published data from a randomized trial in 128 patients after new HTx evaluated the effect of 1-year evolocumab therapy on coronary artery intima–media thickness (IMT), measured by intracoronary ultrasound. Although there was no change in coronary IMT, evolocumab use substantially reduced LDL-C levels by 1.11 mmol/l [95% confidence interval (CI) 0.86–1.37] compared with placebo. Evolocumab treatment was safe in HTx patients and not associated with an increase in adverse events. The trial was not powered to assess the effect of evolocumab on clinical outcomes [12].

The recommended dosage of evolocumab is 140 mg subcutaneously every 2 weeks or 420 mg once a month. Alirocumab is administered at a dose of 75–150 mg every 2 weeks or 300 mg once a month.

Another PCSK9 modulator, inclisiran, is a small interfering RNA (siRNA) silencing the translation of PCSK9 protein. By causing degradation of the mRNA of the PCSK9 protein, the drug inhibits its translation, resulting in an increase in the density of LDL receptors on the liver surface. Although inclisiran plasma concentrations peak at ≈4–6 h after administration, and after 9–48 h this medication disappears from the bloodstream, the therapeutic effect is sustained and persistent, allowing for 6-months intervals between doses [10].

The efficacy and safety of inclisiran have been evaluated in the VICTORION and ORION clinical trials, comprising over 25 studies. The results of phase III randomized controlled trials ORION-9, 10 and 11 in subjects with ASCVD, ASCVD equivalent and familial hypercholesterolaemia showed a 50.7% placebo-corrected reduction in LDL-C by day 510 with no clinically significant adverse effects other than injection site reactions. Moreover, 86.6% of patients reached LDL-C levels <70 mg/dl and 74.6% reached levels <50 mg/dl [33].

The ORION-8 trial, an open-label extension of ORION-9, 10 and 11, involving 3274 patients followed for an average of 36 months, reported an inclisiran-induced mean percentage change in LDL-C of 49.4%. Mild and moderate treatment-emergent adverse events at the injection site occurred in 5.9% of the patients [34]. Inclisiran was also investigated in patients with CKD. The evidence suggests that inclisiran can be safely used in patients with mild, moderate or severe renal impairment without the need for adjustments in dose or the dosing regimen [10].

A recent observational study assessed adherence and persistence at 12 months among patients who newly initiated inclisiran, alirocumab or evolocumab in the longitudinal US Komodo Health database. Inclisiran showed significantly higher adherence and lower rates of discontinuation compared with monoclonal antibodies against PCSK9 at 12 months after initiation, possibly due to convenient dosing of inclisiran [35].

A subanalysis of pooled data from ORION-9, 10 and 11 showed a 25% lower rate of cardiovascular events in the inclisiran arm [13]. Currently a cardiovascular outcome trial [VICTORION-2P (NCT05030428)] is testing inclisiran in individuals with prior myocardial infarction or stroke. Inclisiran has a good safety profile. Treatment-emergent events were comparable in the inclisiran and placebo arms, except for injection site reactions (5% versus 0.7%) and mild to moderate bronchitis [4.3% versus 2.7%; relative risk 1.55 (95% CI 1.09–2.20)].

The US Food and Drug Administration and European Medicines Agency (EMA) registered inclisiran in 2020, intended for subcutaneous administration by a healthcare professional in a dose of 300 mg every 6 months in long-term treatment. However, there is a need for an additional injection after 3 months since initiation.

One report has been published to date on the use of inclisiran in a renal transplant patient diagnosed with coronary artery disease (CAD) and peripheral artery disease receiving IS with everolimus and prednisone. Inclisiran was added to atorvastatin 80 mg/day and ezetimibe with a baseline LDL-C level of 2.46 mmol/l and a GFR of 25 ml/min/1.73 m^2^. The drug was then administered at 3, 6 and 12 months, resulting in a reduction in LDL-C levels to 1.32 mmol/l after 1 year of therapy, with no effect on graft function or everolimus levels [14].

Another promising medication approved in 2020 in the USA and Europe is BA. Its mechanism of action involves blocking adenosine triphosphate citrate lyase, reducing the amount of substrate for cholesterol synthesis and LDL-C concentrations by 30%. BA is a pro-drug requiring activation by an enzyme present in the liver and kidney, but absent in skeletal muscle. This makes the drug indicated for patients with elevated LDL-C levels and statin-induced myalgia. Side effects include an increase in serum uric acid levels, so the drug should be used with caution in patients with gout or predisposed to it. The CLEAR study (NCT02811861) showed that BA combined with a maximum tolerated dose of a statin significantly lowered LDL-C levels and has a good safety profile. The recently completed CLEAR OUTCOMES trial (NCT02993406) of 13 970 statin-intolerant patients at high CV risk showed that BA compared with placebo reduced not only LDL levels by 21%, but also the risk of CV events. The combined treatment of BA and ezetimibe provides a 50% reduction in LDL-C [11].

Although studies on BA in post-transplant patients are lacking, the mechanism of action and lack of interactions with IS drugs indicate that this drug may be a therapeutic option in SOT recipients with a history of statin-induced myalgia. The EMA has approved a combination product of BA and ezetimibe under the name Nustendi (180/10 mg).

### Therapies targeting Lp(a)

Elevated serum Lp(a) concentration is an established risk factor for ASCVD. Currently there are limited treatment options for patients with hyper-Lp(a), including lipoprotein apheresis (LA), able to reduce Lp(a) levels up to 75%. Although PCSK9 modulators can reduce Lp(a) levels by 15–30%, these drugs are not registered for isolated hyper-Lp(a). New medications specifically targeting Lp(a) levels are still be investigated in clinical trials. Parenteral siRNA medications, including olpasiran, lepodisiran and zerlasiran (SLN360), silence the expression of the *LPA* gene encoding apolipoprotein(a) [apo(a)] and reduce serum Lp(a) concentrations up to 100% [36]. Another drug, pelacarsen, an antisense oligonucleotide blocking apo(a) synthesis, has been shown to decrease serum Lp(a) concentrations by 80–90%. The newest drug, muvalaplin, blocking the covalent attachment of apo(a) with the apoB_100_ moiety of LDL particles, lowers serum Lp(a) levels by 65%. However, these drugs are still being evaluated in ongoing CV outcome prospective randomized clinical trials. The first endpoint-driven CV outcome trial [Lp(a)Horizon; NCT04023552] with pelacarsen is estimated to be completed in 2025–2026 [37].

Currently, patients with elevated Lp(a) and ASCVD can be treated with LA. LA was also used successfully in the SOT population. In addition to the decrease in lipids, pleiotropic effects were observed (anti-inflammatory, improvement of rheology, reduction of oxidative stress and oxidized LDLs), which may further reduce the risk of CVD and developing CAV. LA was used in post-HTx patients with CAV, resulting in stabilization of their condition [6, 38].

### Approaches to manage dyslipidaemia after SOT

The European Society of Cardiology (ESC) guidelines indicate SOT recipients as a group have a high risk of CV events, with a general LDL-C goal of <70 mg/dl and a reduction of >50% [3]. However, further estimation of the CV risk category as very high with an LDL-C target of <55 mg/dl should be done based on co-existing comorbidities, as stated below.

In OLTx, pancreas transplant alone (PTA) and LTx recipients, CV risk categories should be identified for dyslipidaemia. The very-high-risk category (target LDL-C <55 mg/dl and reduction of >50%) includes patients with [39] coronary artery disease (percutaneous coronary intervention, coronary artery bypass graft, myocardial infarction), cerebrovascular disease with stroke/transient ischaemic attack, peripheral artery disease, after limb amputation due to ischaemia, aortic aneurysm or atherosclerosis of the internal carotid arteries with stenosis >50%; CKD with a GFR <30 ml/min/1.73 m^2^; CKD with a GFR of 30–59 ml/min/1.73 m^2^ and an albumin:creatinine ratio (ACR) >30 mg/g, diabetes with severe organ damage, GFR <45 ml/min/1.73 m^2^, GFR 45–90 ml/min/1.73 m^2^ and ACR 30–300 mg/g, ACR >300 mg/g or microvascular disease in three locations; Systematic Coronary Risk Evaluation 2 (SCORE2) for the appropriate age group; or familial hypercholesterolaemia with ASCVD or one substantial risk factor.

Patients at high CV risk (target LDL-C <70 mg/dl and reduction of >50%) are those with [39] CKD with a GFR of 30–59 ml/min/1.73 m^2^ and an ACR <30 mg/g; diabetes, including those with target organ complications, who do not meet the criterion of very high risk; SCORE2 ≥5% and <10% for the appropriate age group; significantly increased single risk factors, in particular TC >310 mg/dl and LDL-C >190 mg/dl, or blood pressure ≥180/110 mm Hg; or familial hypercholesterolaemia.

In HTx and KTx patients, statins are recommended regardless of LDL-C levels (ESC Class IA, ISHLT Class IA, KDIGO Class 2A) [20, 21].

In OLTx, PTA and LTx regardless of the CV risk category, if LDL-C levels are >190 mg/dl, statins should be started immediately and lifestyle modified.

Table 2 presents the published randomized controlled trials on statin therapy in SOT recipients.

**Table 2:** Randomized clinical trials on lipid-lowering agents in SOT recipients.

Study	Country	Sample size, n	Statin	Transplanted organ	Time from Tx^a^	Age (years)	Male (%)	DM (%)	HTN (%)	BMI (kg/m^2^)	eGFR (ml/min/1.73 m^2^)	LDL-C (mmol/l)	CVD (%)	CNI (%)	mTORi (%)	Follow-up (months)^a^
2008 Seron (PMID: 18622282)	Spain	89	Fluvastatin (80 mg)	Kidney	Post-transplant	42	57.3	0	NR	NR	NR	NR	0	CsA (100)	0	72
2004; Fellström- ALERT (PMID: 15458450)	International	2102	Fluvastatin (40 mg)	Kidney	62.4 months	49.8	66	18.8	74.9	25.8	60.3	4.1	23.5	CsA (100)	NR	61.2
2001; Holdaas (PMID: 12814712)	International	364	Fluvastatin (40 mg)	Kidney	Post-transplant	48.4	71.4	12.4	NR	NR	NR	2.98	NR	CsA (100)	0	3
1999; Lepre (PMID: 10617243)	Australia	49	Simvastatin (10 mg)	Kidney	>12 months	51.4	34.7	14.3	77.6	NR	NR	4.63	NR	CsA (57.1)	0	9
1996; Katznelson (PMID: 863337)	USA	48	Pravastatin (20 mg)	Kidney	7 days	47.3	69	NR	NR	NR	NR	NR	NR	CsA (100)	0	4
1994; Arnadotirr (PMID: 7991041)	USA	40	Simvastatin (10 mg)	Kidney	50 months	51.5	65	20	NR	25.3	NR	5.45	NR	CsA (100)	0	5
2005; Holdaas ALERT (PMID: 16303007)	International	1652	Fluvastatin (40 mg)	Kidney	62.4 months	48.5	66.3	16.9	74.6	25	60.3	4.1	23.5	CsA (100)	0	72
2003; Holdaas ALERT (PMID: 12814712)	International	2102	Fluvastatin (40 mg)	Kidney	62.4 months	49.8	66	18.8	74.9	25.8	60.3	4.1	23.5	CsA (100)	0	72
2001; Santos (PMID: 11267254)	Brazil	67	Simvastatin (10 mg)	Kidney	60.5 months	43.3	44.8	NR	NR	NR	65.9	4.67	NR	CsA (77.6)	0	57.5
2001; Kasiske (PMID: 11477342)	USA	105	Simvastatin (10 mg	Kidney	Post-transplant	46.5	59	NR	NR	NR	NR	NR	NR	CsA (100)	0	3
2024 Broch (PMID: 38934968)	International	110	Evolocumab (420 mg)	Heart	4–8 weeks	52.5	74.5	19	32.7	24.5	65	2.56	29	TAC (88.1), CsA (10)	(20.9)	12
2019 Nykänen (PMID: 31352795)	Finland	84	Simvastatin (80 mg)	Heart	Post-transplant	57.5	73.8	11.9	13.1	25.4	55	NR	22.6	TAC (80.9), CsA (6.7)	0	24 h
2000 Magnani (PMID: 10930822)	Italy	39	Atorvastatin (10–20 mg), pravastatin (20–40 mg)	Heart	25 months	56	87.2	10.2	NR	NR	NR	4.42	41	CsA (100)	0	4
2009 Soveri ALERT (PMID: 19594771)	International	1706	Fluvastatin (40 mg)	Kidney	62.4 months	49	66	0	74.5	NR	47.5	4.2	10.8	CsA (97.6)	NR	7.4 years
2004 O'Rourke (PMID: 15093987)	USA	79	Fluvastatin (40 mg/day)	Heart	3.6 ± 2.99 years	52	83	ND	ND	29.0	ND	4.54	58	ND	ND	1 year

^a^Median values.

DM: diabetes mellitus; HTN: hypertension; BMI: body mass index; mTORi: mammalian target of rapamycin inhibitor; TAC: tacrolimus; Tx: transplantation; NR: not reported.

**Figure 1:**
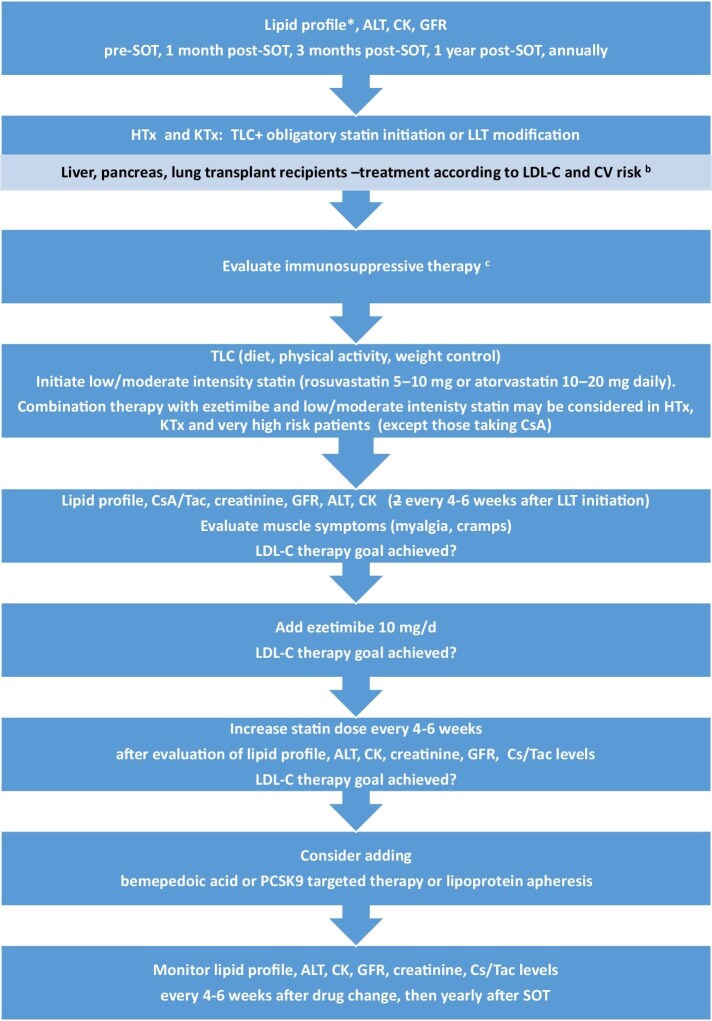
Management of hypercholesterolaemia in SOT recipients. (**a**) TG >400 mg/dl: measure direct LDL-C. (**b**) LDL-C >190 mg/dl: IS evaluation, TLC and statin initiation; LDL-C 115–189 mg/dl in a high- or very-high-risk patient: IS evaluation, TLC 3 months, after 3 months of TLC when LDL-C is >100 mg/dl initiate statin treatment; TG >500 mg/dl: IS evaluation (GC, mTOR inhibitor), diet evaluation, exclude alcohol use, diabetes, kidney failure (GFR, proteinuria). (**c**) Consider switching CsA to tacrolimus or reducing the CsA dose, adding/increasing the dose of mycophenolate mofetil, withdrawal of or reducing the doses of corticosteroids and mTOR inhibitors (when TG >500 mg/dl). TLC: therapeutic life changes; CK: creatine kinase.

A step-by-step approach using statins alone initially is proposed for SOT recipients. If the LDL-C treatment goals are not achieved, ezetimibe should be added. In this strategy the third-line treatments are therapies targeting PCSK9 (Fig. 1).

The data from the SANTORINI study (NCT04271280) showed that LDL-C goals cannot be achieved in >70% of patients. The reasons could be the non-adherence to statins and the time-consuming stepwise approach, which requires >2–3 months to assess the effectiveness of each step. Combination therapy compared with monotherapy improved LDL-C management and goal achievement (39.4% versus 25.5%).

Early achievement of low LDL-C levels is crucial in patients at high or very high CV risk. Therefore, an ESC/European Atherosclerosis Society expert statement proposed a strategy of ‘strike early and strong’ using ezetimibe in combination with a high-intensity statin. The 2023 ESC guidelines on acute coronary syndromes (ACSs) indicated that initiating a statin in combination with ezetimibe may be considered in statin-naïve ACS patients with high LDL-C levels, indicating difficulties in reaching the targets using statin monotherapy. The lipid profile should be reassessed 4–6 weeks after each treatment or dose adjustment to determine safety issues and achievement of treatment goals and to adjust treatment accordingly. Patients who do not reach their LDL-C targets despite statin and ezetimibe therapy should start PCSK9 inhibitor treatment (Fig. 1). This strategy utilizing combination therapy with ezetimibe and statin from the start allows one to safely and efficiently reduce LDL-C levels by >50% [9].

A similar algorithm may be considered in SOT patients with a high or very high risk of CV events. It is worth considering initiating combined treatment with ezetimibe and a low-/moderate-intensity statin in HTx and KTx patients and in SOT patients with a very high CV risk (established ASCVD, diabetes with target organ damage, CKD with a GFR <30 ml/min/1.73 m^2^).

**Figure 2:**
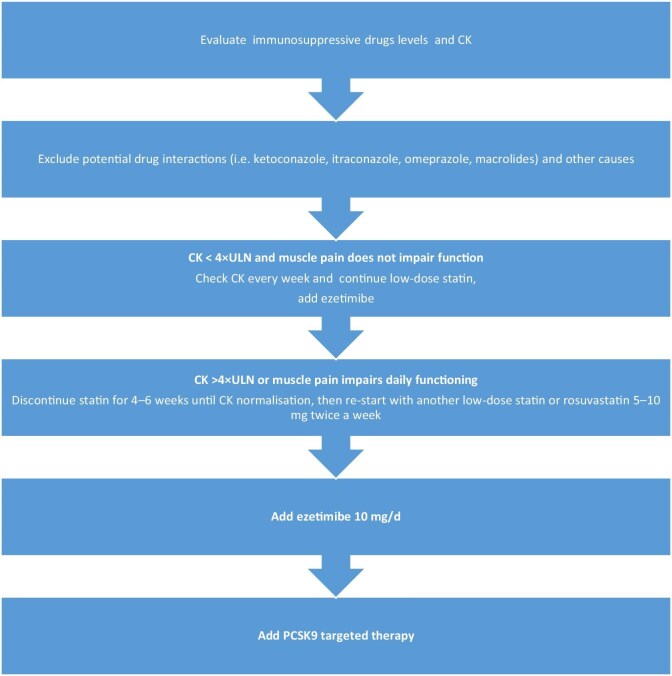
Management of statin-associated muscle symptoms in SOT recipients. CK: creatine kinase.

SOT patients could start with a low/moderate dose of statin in combination with ezetimibe 10 mg/day. Based on data from the RACING trial, it could be a fixed-dose combination of low/moderate statin with ezetimibe. Only SOT patients treated with CsA should start with a low/moderate statin, then ezetimibe (5 mg) should be administrated after evaluation of LDL-C levels. BA and PCSK9 targeted therapy should be added if combined treatment with maximally tolerated statin and ezetimibe does not provide sufficient LDL-C reduction, or in case of statin intolerance. Fig. 2 presents an algorithm in case of statin intolerance or adverse events considering the immunosuppressive regimen and creatine kinase levels.

Table 3 presents the impact of lipid-lowering agents on LDL-C and TG [40].

**Table 3.** The impact of lipid lowering agents on LDL-C and TG [40].

		
Lipid lowering medications	LDL-C reduction	TG reduction
		
Ezetimibe 10 mg	20%	
Rosuvastatin 5–10 mg/day Atorvastatin 10–20 mg/day Pitavastatin 4 mg/day Simvastatin 20–40 mg/day Pravastatin 40 mg/day Lovastatin 40 mg/day Fluvastatin 80 mg/day Pitavastatin 1–2 mg + Ezetimibe 10 mg/day Pitavastatin 1–2 mg + Ezetimibe 10 mg/day Simvastatin 10–20 mg/day + Ezetimibe 10 mg/day Pravastatin 20 mg/day + Ezetimibe 10 mg/day Lovastatin 20 mg + Ezetimibe 10 mg/day Fluvastatin 40 mg + Ezetimibe 10 mg/day Bempedoic acid 180 mg/day + Ezetimibe 10 mg/day	30-50%	
Atorvastatin 40–80 mg/day Rosuvastatin 20–40 mg/day	50–60%	
Rosuvastatin 5–10 mg/day + Ezetimibe 10 mg/day Atorvastatin 10–20 mg/day + Ezetimibe 10 mg/day Pitavastatin 4 mg + Ezetimibe 10 mg/day Simvastatin 20–40 mg/day + Ezetimibe 10 mg/day Pravastatin 40 mg/day + Ezetimibe 10 mg/day Lovastatin 40 mg/day + Ezetimibe 10 mg/day Fluvastatin 80 mg/day + Ezetimibe 10 mg/day	50–60%	
Atorvastatin 40–80 mg/day + Ezetimibe 10 mg/day Rosuvastatin 20-40 mg+ Ezetimibe 10 mg/day	65%	
PCSK9 inhibitors (alirocumab, evolocumab)	45-65%	
Inclisiran	45-50%	
High intensive statin + ezetimibe plus PCSK9 targeted therapy (alirocumab, ewolocumab, inclisiran)	80-85%	
Fibrates		25-50%
Omega 3 acids		20–30%

It should be noted that alirocumab, evolocumab, inclisiran and BA have a strong safety profile compared with conventional LLT and a low risk of interactions with IS medications. BA, due to its mechanism of action, can be a therapeutic option in SOT recipients with a history of statin-induced myalgia. Alirocumab and evolocumab, fully humanized monoclonal antibodies against PCSK9, administrated biweekly/once monthly can reduce LDL-C levels by 60% and have little potential for interactions with IS. Inclisiran injected by healthcare professionals every 6 months has a similar rate of treatment-emergent adverse events as placebo. Moreover, these drugs may have promising CV outcomes in SOT patients.

### Management of dyslipidaemia in heart transplantation

Lipid disorders in HTx patients are a common complication with a multifactorial aetiology, leading not only to coronary artery atherosclerosis but also to CAV, which is currently the predominant cause of mortality among HTx patients [20]. If the donor is older, the presence of both processes in the graft should be expected, especially in recipients >40 years of age [2].

Elevated LDL-C levels during the first year after HTx were associated with a severe course of CAV (assessed by intravascular ultrasound), while maintaining LDL-C at <100 mg/dl reduced the risk of developing CAV. Thus CAV prevention after HTx includes rigorous control of hyperlipidaemia and other CV risk factors (Class IC). [20]

Increased Lp(a) levels are also an independent predictor of CAV development. For every Lp(a) concentration increase of 10 mg/dl, the risk of developing CAV increases by 26%. Lp(a) concentration and the TG:HDL-C ratio were substantially related with fibrotic plaque in HTx recipients, representing an additional target for CAV prevention.

Statin therapy after HTx has become an integral part of patient care. Along with LDL lowering and pleiotropic effects, statins also improve endothelial function in the coronary arteries. The Canadian Cardiovascular Society recommends statins in HTx patients regardless of lipid levels. Also, the ISHLT recommends that every post-HTx patient should begin treatment with statins, regardless of baseline lipid levels (Class 1A) [20, 41].

In the post-HTx paediatric population, the ISHLT recommends routine use of statins in children >10 years of age, as well as in younger recipients with hyperlipidaemia, CAV or those after retransplantation (Class IIC). However, a recently published study showed no obvious benefit of statin treatment in improving CV prognosis in paediatric HTx recipients [42].

The goals of therapy for HTx patients are not clearly defined in the ESC guidelines and depend on the CV risk category. Thus, for patients in the very high CV risk category, the goal of therapy is to reduce LDL-C by at least 50% and <55 mg/dl. The target LDL-C in patients in the high CV risk category is <70 mg/dl. The published data show that a concentration of LDL-C <100 mg/dL should be maintained in post-HTx patients and more aggressively lowered for CAV (Class IIa, level of evidence: C).

Statin treatment should be started at lower doses due to the risk of drug interactions with CNIs and toxicity, while verifying IS therapy [3, 20].

Results of previous studies suggest the safety of combining tacrolimus with statins. The preferred statins after HTx are rosuvastatin, pitavastatin and pravastatin, due to a lower risk of toxicity and interactions. The initial and maximum daily doses for rosuvastatin are 5–10 and 20 mg, respectively, and for pravastatin 20 and 40 mg, respectively. Statins improve the prognosis and survival of HTx patients. Simvastatin added to a dietary intervention versus diet alone showed a significant reduction in LDL-C, a reduction in CAV risk and improvement in 4-year prognosis. A meta-analysis of nine randomized and observational studies involving 2295 HTx recipients treated with statins showed a significant reduction in overall mortality in the statin-treated group compared with HTx patients without statin therapy [odds ratio 0.26 (95% CI 0.20–0.35), *P* < .0001]. Statin therapy reduced the incidence of CAV and major graft rejection by 67% and 63%, respectively [1].

Also important in LLT after HTx is the intensity of statins used, i.e. their type and dose, defined as low, moderate or high. Lower statin intensity, a history of ischaemic cardiomyopathy and acute rejection were significantly associated with the primary composite endpoint, defined as heart failure, myocardial infarction, revascularisation and all-cause mortality. The greatest benefit came from the early use of higher-intensity statins after HTx.

The timing of statin initiation is also an important consideration. Statin administration in the pre-HTx period reduced the risk of graft dysfunction by 65% and in-hospital mortality by 73%. Patients receiving statins before HTx had significantly lower mortality at 1 and 5 years [43].

Ezetimibe has also been used in the treatment of HTx recipients with hypercholesterolaemia. A combination of ezetimibe with a low-dose statin can safely and effectively reduce LDL-C without the risk of toxicity. Ezetimibe can also be used in monotherapy in statin-intolerant patients, as well as in children with genetically determined hypercholesterolaemia [8].

Monoclonal antibodies against the PCSK9 protein (evolocumab, alirocumab) in HTx recipients have been shown to safely and efficiently lower LDL-C levels without clinically significant interactions with IS drugs. PCSK9 inhibitors can be used in post-HTx patients with severe hyperlipidaemia as an adjunct to statins, but also in those with intolerance to statins (ISHLT Class IIb, level of evidence: B) [20]. The lipid profile should be monitored twice a year in adults after HTx (Class I, level of evidence: C) [20].

In conclusion, LLT initiated early after HTx contributes to reducing cardiac allograft rejection and CAV rates and improving short- and long-term prognoses. Statin initiation should be considered before HTx due to the observed favourable effect of statins on 1- and 5-year survival rates.

### Management of dyslipidaemia in kidney transplantation

Dyslipidaemia in KTx recipients mostly results from IS therapy but can also be the result of prior renal insufficiency, haemodialysis or peritoneal dialysis therapy. Low physical activity, poor diet, weight gain, insulin resistance and de novo diabetes may also contribute to dyslipidaemia after KTx. CVD is common in KTx recipients. In stages 3 and 4–5 of CKD and after KTx, CVD is the predominant cause of death [35]. In CKD patients undergoing KTx, the risk of ASCVD can be estimated based on the GFR and high or very high CV risk categories [44].

The most prevalent dyslipidaemia in KTx recipients is hyperlipidaemia, with elevated concentrations of TG, TC and LDL-C. HDL-C levels may be reduced or within normal range. It is estimated that up to 40% of KTx recipients experience a CV event within 36 months after organ transplantation and dyslipidaemia in KTx recipients also contributes to IF/TA, resulting in kidney allograft failure [44].

Statins in KTx recipients are first-line lipid-lowering medications. Fluvastatin has been shown to reduce the incidence of secondary composite endpoints (combined cardiac death or non-fatal myocardial infarction, cerebrovascular events, non-CV death, all-cause mortality and graft loss or doubling of serum creatinine) in the prospective Assessment of Lescol in Renal Transplantation (ALERT) trial [45]. Statin therapy in KTx recipients without CAD can reduce the risk of CV events. Instead of high doses of potent statins (atorvastatin 40–80 mg/day and rosuvastatin 20–40 mg/day), a moderate-intensity statin in combination with ezetimibe should be considered. Nevertheless, published data show that statins were administrated in only 41.6% of KTx recipients and hyperlipidaemia was poorly controlled [44].

In addition to statins and ezetimibe, fibrates also found their place in the KDIGO guidelines. However, fibrate interactions with statins and other medications are often observed in KTx recipients due to decreased GFR and the high number of medications used.

After KTx, a 35% reduction in Lp(a) concentration is also observed [38]. A greater decrease in Lp(a) levels was observed in those with higher pre-KTx concentrations and in those with large apo(a) isoforms. Interestingly, patients <35 years of age with small apo(a) isoforms showed significantly shorter allograft function compared with those with large isoforms, and this was independent of gender, IS therapy or human leucocyte antigen incompatibility [46].

### Management of dyslipidaemia in liver, pancreas and lung transplantation

No specific recommendations have yet been published on the management of dyslipidaemia in patients undergoing OLTx, PTA and LTx. However, data indicate that statins are beneficial in OLTx, PTA and LTx patients, reducing the risk of allograft rejection, prolonging allograft function and reducing the incidence of post-operative complications [1].

Although lung transplant recipients have a high incidence of CAD, even in the absence of CV risk factors, optimally managed CAD is not associated with a worse prognosis. Retrospective analysis of 172 LTx recipients revealed an association between pre-transplant TC levels and vascular events (dialysis initiation, cerebrovascular incident, myocardial infarction). The TC:HDL ratio was correlated with mortality after LTx [47]. In another retrospective study that included 785 LTx recipients, statin use for a minimum of 2 weeks in the first year after transplantation was associated with a 70% reduction in the risk of invasive aspergillosis [20].

Dyslipidaemia in OLTx recipients is a common complication and, interestingly, can be caused (rarely) by transplantation of the liver from a donor carrying variants in the *LDLR* and *LPA* genes. Huang *et al.* [48] reported new-onset dyslipidaemia in 70.2% liver recipients during a follow-up period of ≈2 years. A close correlation between post-transplant dyslipidaemia and overweight and the pre-transplant lipid profile was found. Overall survival and tumour-free survival were substantially lower in patients with dyslipidaemia compared with those without lipid disorders.

Another study showed the association between new-onset hypercholesterolaemia within 1 year following a living-donor OLTx and long-term CV outcome [hazard ratio (HR) 2.77 (95% CI 1.16–6.61), *P* = .02] and graft failure [HR 3.76 (95% CI 1.97–7.17), *P* < .001] [49]. The use of mTOR inhibitors in OLTx recipients with advanced stages of hepatocellular carcinoma independently increased the development of hypercholesterolaemia. Moreover, statins can inhibit the recurrence of hepatocellular carcinoma and decrease mortality by preventing hepatic decompensation as well as the progression of hepatic fibrosis.

There is a paucity of publications related to metabolic follow-up evaluation and the management of diabetic patients after PTA/simultaneous pancreas–kidney transplantation (SPK). The majority of PTA and SPK recipients have dyslipidaemia before transplantation, due to poorly controlled diabetes, and most of them due to diabetic kidney disease [1]. Compared with preoperative levels, patients who had SPK show improvements in their lipid profile, while those after PTA remain with stable lipid levels [50]. In the early post-transplant period, high doses of IS can aggravate hyperlipidaemia. Lipid levels tend to stabilize during first year. Steroid withdrawal regimens have been related to a lower prevalence of hyperlipidaemia after PTA/SPK transplantation. Tacrolimus appears to have fewer adverse effects on lipids than CsA. The improvement in overall mortality, stroke, kidney function and toxicity outcomes in PTA and SPK recipients related to statins is uncertain. The use of statins in PTA patients may lead to improved outcomes. Whether it is due to CV protection or other factors unrelated to lipid lowering remains unclear.

## CONCLUSIONS

Lipid disorders in the pre- and post-transplant period are often underdiagnosed and undertreated. Interactions between diet, overweight, impaired glucose metabolism, genetic predisposition and IS therapy trigger and aggravate post-transplant dyslipidaemia. Individualization of dietary interventions, IS therapy and LLT is needed to weigh the risk of acute graft rejection and efficient and safe lipid control. Nevertheless, data on the management of lipid disorders in SOT patients are very limited. The common problem of statin non-adherence, potential interactions with IS as well as concerns related to polypharmacy impact statin use in SOT recipients. Considering an early initiation of combination therapy with a low-/moderate-intensity statin and ezetimibe, novel parenteral PCSK9 modulators and BA can provide safe and efficient lipid management, translating into a decrease in the rate of CV events, allograft rejection and vasculopathy. The broader use of newer lipid-lowering agents injected once monthly or every 6 months could improve adherence and increase the achievement of LDL-C targets.

An important consideration is also the timing of LLT initiation. HTx and KTx patients of very high or high CV risk requiring LLT regardless of lipid levels could benefit the most from an early efficient novel LLT. Another group, SOT recipients with coexisting ASCVD requiring an LDL-C decrease to <55 mg/dl, could be also benefit from novel lipid-lowering agents.

Moreover, a structured protocol involving a multidisciplinary approach is necessary for effective lipid management in SOT patients.

Further clinical trials and registries powered to assess the effect of interventions on clinical outcomes are needed in SOT patients. To conclude, we call for further studies in SOT recipients to determine potential benefits of initial combination therapy with ezetimibe and a low/moderate dose of statin; the safety, efficacy and benefits of novel lipid-lowering drugs (evolocumab, alirocumab, inclisiran, BA) in prospective randomized trials; and the optimal lipid-lowering goals depending on the transplanted organ and CV risk category.

## Data Availability

The data are included in the article. Further inquiries can be directed to the corresponding author.
